# Genes That Influence Swarming Motility and Biofilm Formation in *Variovorax paradoxus* EPS

**DOI:** 10.1371/journal.pone.0031832

**Published:** 2012-02-21

**Authors:** Michael J. Pehl, William David Jamieson, Karen Kong, Jessica L. Forbester, Richard J. Fredendall, Glenn A. Gregory, Jacob E. McFarland, Jessica M. Healy, Paul M. Orwin

**Affiliations:** 1 Department of Biology, California State University San Bernardino, San Bernardino, California, United States of America; 2 Department of Biology and Biochemistry, University of Bath, Bath, United Kingdom; 3 Loma Linda University School of Dentistry, Loma Linda, California, United States of America; 4 UCSF School of Pharmacy, University of California San Francisco, San Francisco, California, United States of America; 5 UCD Department of Public Health Sciences 5215 VM3A, Davis, California, United States of America; University of Padova, Medical School, Italy

## Abstract

**Background:**

*Variovorax paradoxus* is an aerobic soil bacterium associated with important biodegradative processes in nature. We use *V. paradoxus* EPS to study multicellular behaviors on surfaces.

**Methodology:**

We recovered flanking sequence from 123 clones in a Tn5 mutant library, with insertions in 29 different genes, selected based on observed surface behavior phenotypes. We identified three genes, Varpa_4665, Varpa_4680, and Varpa_5900, for further examination. These genes were cloned into pBBR1MCS2 and used to complement the insertion mutants. We also analyzed expression of Varpa_4680 and Varpa_5900 under different growth conditions by qPCR.

**Results:**

The 29 genes we identified had diverse predicted functions, many in exopolysaccharide synthesis. Varpa_4680, the most commonly recovered insertion site, encodes a putative N-acetyl-L-fucosamine transferase similar to WbuB. Expression of this gene *in trans* complemented the mutant fully. Several unique insertions were identified in Varpa_5900, which is one of three predicted *pil*Y1 homologs in the EPS genome. No insertions in the two other putative *pil*Y1 homologs present in the genome were identified. Expression of Varpa_5900 altered the structure of the wild type swarm, as did disruption of the chromosomal gene. The swarming phenotype was complemented by expression of Varpa_5900 from a plasmid, but biofilm formation was not restored. Both Varpa_4680 and Varpa_5900 transcripts were downregulated in biofilms and upregulated during swarming when compared to log phase culture. We identified a putative two component system (Varpa_4664-4665) encoding a response regulator (*shk*R) and a sensor histidine kinase (*shk*S), respectively. Biofilm formation increased and swarming was strongly delayed in the Varpa_4665 (*shk*S) mutant. Complementation of *shk*S restored the biofilm phenotype but swarming was still delayed. Expression of *shk*R *in trans* suppressed biofilm formation in either genetic background, and partially restored swarming in the mutant.

**Conclusions:**

The data presented here point to complex regulation of these surface behaviors.

## Introduction

*Variovorax paradoxus* is an aerobic β-proteobacterium in the family Comomonadaceae present in diverse environments [1], initially identified as a Knallgas bacterium. True to its name, *V. paradoxus* strains have been isolated that can perform many important biotransformations, including atrazine degradation [2], nitrotyrosine assimilation [3], mineralization of acyl-homoserine lactone signals [4]. Strains of *V. paradoxus* have been identified as plant growth promoting rhizobacteria (PGPR), with the growth promotion attributed to either the hydrogen gas oxidation growth strategy of *V. paradoxus* [5] or to the production of ACC deaminase and the mineralization of aminocyclopropane [6]. Recent work has also shown this organism associated with methylotrophy in the human oral cavity [7]. Relatively little has been published on the basic physiology of *V. paradoxus*, even with this ample basis for interest. Recently, our laboratory has examined the interactions of this organism with abiotic surfaces [8], and the genomes of two strains have been sequenced [9].

Bacterial growth on surfaces is clearly an important and rapidly expanding area of research. Biofilms are thought to be the dominant form of microbial growth in many environments, ranging from desert crusts and marine snow to biofilms on medical devices [10]. Surface motility is also clearly important for survival, as evidenced by the wealth of different motility strategies present in the microbial world [11]. Just among the prokaryotes in the soil, one may observe sliding, gliding, twitching, and swarming motility, all of which may serve the purpose of propelling the microbes toward nutrients and away from adverse conditions. Swarming motility, which is present and demonstrably relevant in several pathogenesis systems, has been particularly well studied with respect to its relationship to biofilm formation [12]. The relationship between surface adhesion, sessile growth, and motility on surfaces has become clearer and more relevant to nearly every facet of microbiology [13].

Since Griffith's experiments with pneumococci [14], colony morphology has been a useful indicator of strain variation. The complex communities of bacteria forming a colony on an agar plate can also be used as a model system for studying growth physiology [15]. Strong correlations between colony morphology and biofilm phenotypes have been used for molecular genetic analysis in *Vibrio cholerae* [16], *Pseudomonas aeruginosa* [17], and *Staphylococcus aureus* [18]. Studies using *P. aeruginosa* as a model have identified a complex coordinated regulatory system controlling swarming and biofilm formation, which includes regulatory RNA molecules, quorum sensing, transcription factors, and the global regulatory second messenger cyclic-di-GMP [19] [20].

Transposon insertion mutants were screened by colony morphology and evaluated for altered interactions with surfaces. A total of 123 mutants were identified and sequenced in this manner. A diverse set of mutants was recovered, with the two most common insertions in Varpa_4680, encoding a glycosyl transferase enzyme, and Varpa_5900, encoding a *pil*Y1 ortholog. We analyzed the expression of both of these genes under different growth conditions using qPCR with an exogenous internal reference standard (cite alvarez cohen), and attempted to complement the insertions in each gene. The Varpa_4680 insertion was complemented by expression *in trans*, when tested in biofilm and swarming assays. Varpa_5900 insertion resulted in altered biofilm phenotype even when the wild-type gene was present, although the rate of swarming was restored to normal levels.. Expression of both genes was reduced during biofilm formation and increased in the swarm compared to expression during log phase growth. Finally, a regulatory locus encoding a putative two-component regulatory system, *shk*RS (Varpa4664-5) was identified. The interruption of the sensor increased biofilm levels and strongly delayed swarming motility. Reintroduction of the *shk*S gene resulted in complementation of the biofilm but not the swarming phenotype, while introduction of the *shk*R gene suppressed both biofilm production and swarming rate in either the *shk*S mutant wild type background. These data suggest that *V. paradoxus* EPS responds to its environment through coordinated expression of genes involved in exopolysaccharide production and type IV pilus formation, among others, leading to switching between sessile and motile growth phenotypes depending on the conditions.

## Results

### Mutant Screen

Because colony morphologies were related to nutrient levels, a library of Tn5 insertions were screened on two different nutrient levels (0.5 g/L, 2.5 g/L) for colony morphology variation. After toothpick replicate analysis, approximately 500 stable variants were examined using the static 96 well microtiter biofilm test [21]. After repeated screening, rescue cloning and subsequent sequencing, 123 mutant clones were identified. The mutants identified fell into a number of categories, with varying frequencies of recovery. Insertions in to 29 genes with putative known functions are listed in **Table 1**. Among the mutants identified are insertions in putative exopolysaccharide synthesis operons, type IV pilus (tfp) formation, type VI secretion, general secretion (sec-dependent), as well as several putative regulatory loci, factors in autotrophic growth, as well as many hypothetical proteins of unknown function. Two genes were recovered repeatedly under both assay conditions with multiple unique insertions (bold in **Table 1**). These two genes, Varpa_4680 (16 unique insertions) and Varpa_5900 (11 unique insertions), encode a putative L-fucosamine transferase involved in exopolysaccharide synthesis (*wbu*B) and a putative type IV pilus tip adhesion (*pil*Y1), respectively.

**Table 1:** Mutants from Colony Morphology Screens.

*Function*	*Gene #*	*Gene name*	*Nearest homology*	*Biofilm*	*Swarm*
EPS/LPS	**4680**	***wbu*** **B**	***Sulfurimonas***	**Up**	**Down**
	4681	*gal*E	*Vibrio cholerae*	Up	No effect
	4682	*rfa*G	*Rubrobacter*	Up	Up
	4679	Epimerase	*Polaromonas* JS666	Up	No effect
	4678	Cap8m	*Polaromonas*	Up	Down
	4684	Cap5h	*Vibrio angustum*	Up	n.d.
	4686	*wbp*E	*Polaromonas*	Up	Down
	4674	*eps*B	*Polaromonas*	Down	No effect
	4685	Polysacch synthesis	*Roseiflexus*	Up	Down
	1089	Btwn hyp and *eps*A	*V. paradoxus* S110	Up	Up
	1090	*epsA*	*V. paradoxus* S110	Up	Up
	1093	*exo*Y	*V. paradoxus* S110	Up	Down
	1094	Unk in EPS locus	*Cupriavidus taiwanenis*	Down	No effect
	780	Glycosyltransferase	*Stenotrophomonas maltophilia*	Up	n.d.
Signal	5483	*tet*R regulator	*Polaromonas*	Down	n.d.
transduction	**4665**	***shk*** **S –sensor histidine kinase**	***V. paradoxus*** ** S110**	**Up**	**Down**
	1030	BH2933	*Bacillus halodurans*	Up	N.d.
Surface	**5900**	**PilY1**	**Several species**	**Mixed**	**Mixed**
proteins	2155	Filamentous Haemagglutinin OMP	*Comamonas testosteroni* HecA	Up	No effect
	4377	YaeC lipoprotein	*V. paradoxus* S110	Up	No effect
T6SS	5525	TM protein in T6SS region	*Xanthomonas oryzae*	Down	n.d.
	5533	EvpB type protein	*Cupriavidus*	Down	No effect
Autotrophy	3601	RuBISCO	*Methanocaldococcus*	Down	Down
	3603	Phosphoribulo kinase	*Methylacidiphilum infernorum V4*	Down	No effect
Other	4383	SecA preprotein translocase	*Pseudomonas putida*	Up	Down
	1032	Phosphoglycerate mutase	*V. paradoxus* S110	Down	Down
	2156	ShlB type polypeptide transport	*V. paradoxus* S110	Up	Up
	2158	ShlB type polypeptide transport	*Ralstonia solanacearum*	Up	n.d.
	5158	TadE protein	*V. paradoxus* S110	Down	No effect
	126	Hypothetical	*Ruegeria pomeroyi*	Up	Down

### Swarming motility assay

Based on previous experimental evidence in other systems relating swarming motility to biofilm formation, we examined the responses of our mutant obtained from the library in a standardized swarming assay. We used plating conditions based on our previous work with *V. paradoxus* EPS [8]. We identified a number of mutants with defects in swarming motility as well as biofilm. As shown in **Table 1**, the vast majority of the mutants had alterations in both behaviors. The most drastic phenotype observed during this experiment was in a mutant interrupted in Varpa_4665, encoding a putative sensor histidine kinase (**Table 1**, bold). The disruption of this gene resulted in the inhibition of swarming during the 48 h test period. Subsequent experiments (not shown) demonstrated that after 7 d some swarming could be observed. This gene has therefore been named *s*urface-behavior *h*istidine *k*inase sensor (*shk*S). We identified a number of gene interruptions that had the inverse effect on swarming and biofilm formation that has been observed in other organisms [20], but also mutations that resulted in different relationships between these two phenotypes (**Table 1**). Additional variation in swarm edges and overall swarm morphology were catalogued as well (not shown).

### Varpa_4680 and Varpa_5900 complementation

**Figure 1:**
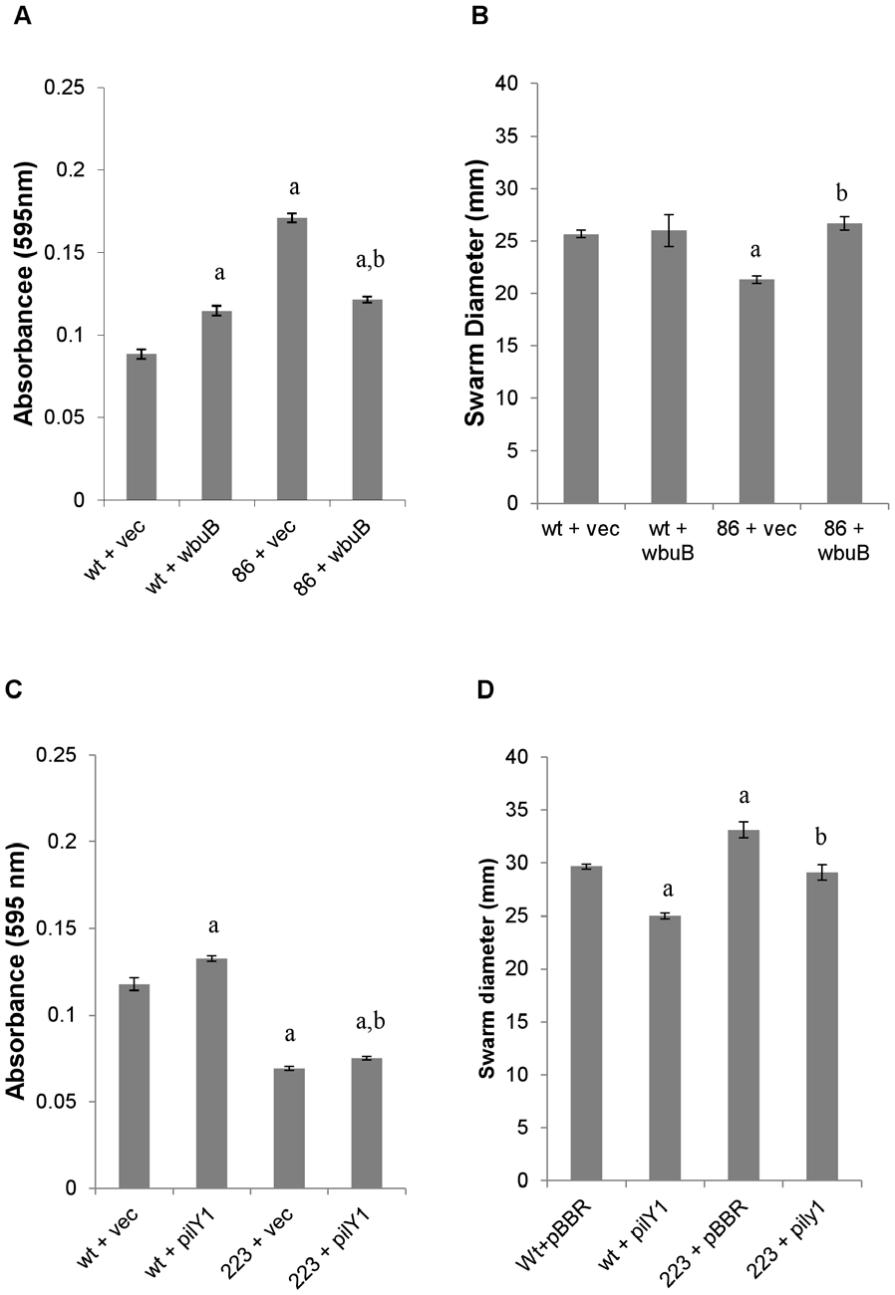
Analysis of surface behaviors to determine efficacy of Varpa_4680 (*wbu*B) and Varpa_5900 (*pil*Y1) complementation. Biofilm levels after 24 h incubation and swarm diameters at 48 h for wt and mutant strains expressing either *wbu*B (A, B) or *pil*Y1 (C, D) *in trans* compared to vector controls. 86 = Varpa_4680::Tn5, 223 = Varpa_5900::Tn5. Error was computed as +/−SEM. All p-values were calculated using the student's unpaired T-test. For all panels a = p<0.01 compared to wt+vec, b = p<0.01 compared to 86+vec (A,B) or 223+vec (C,D).

**Figure 2:**
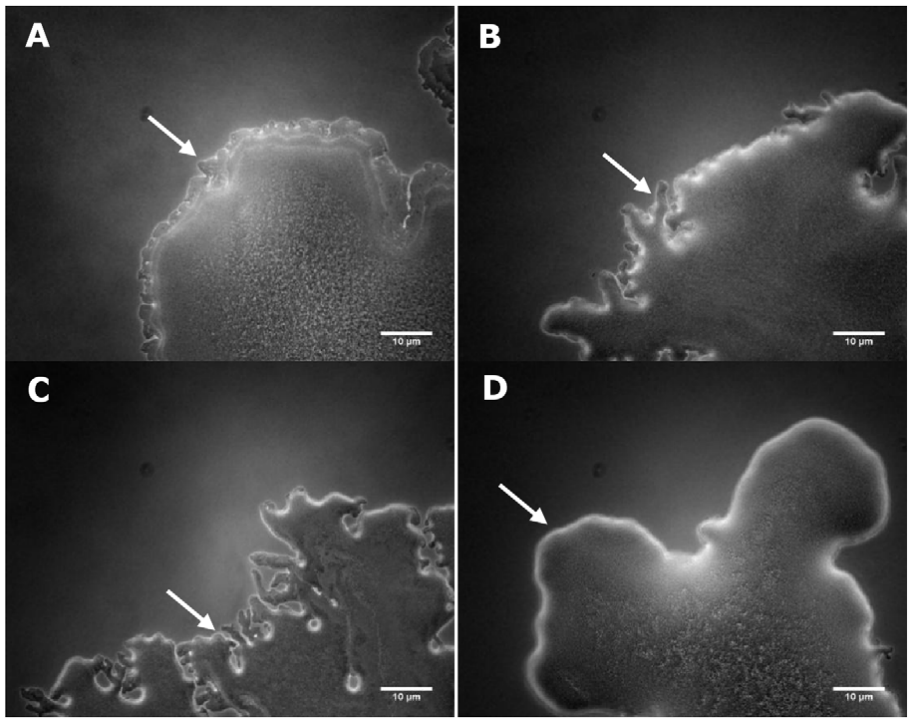
Swarm edges at 24 h on 0.5% FW plates+25 µg/ml Km were photographed using a 10× phase contrast lens (scale bar = 10 microns). A) wt+vec, B) wt+*wbu*B, C) mut86+vec, D) mut86+*wbu*B.

**Figure 3:**
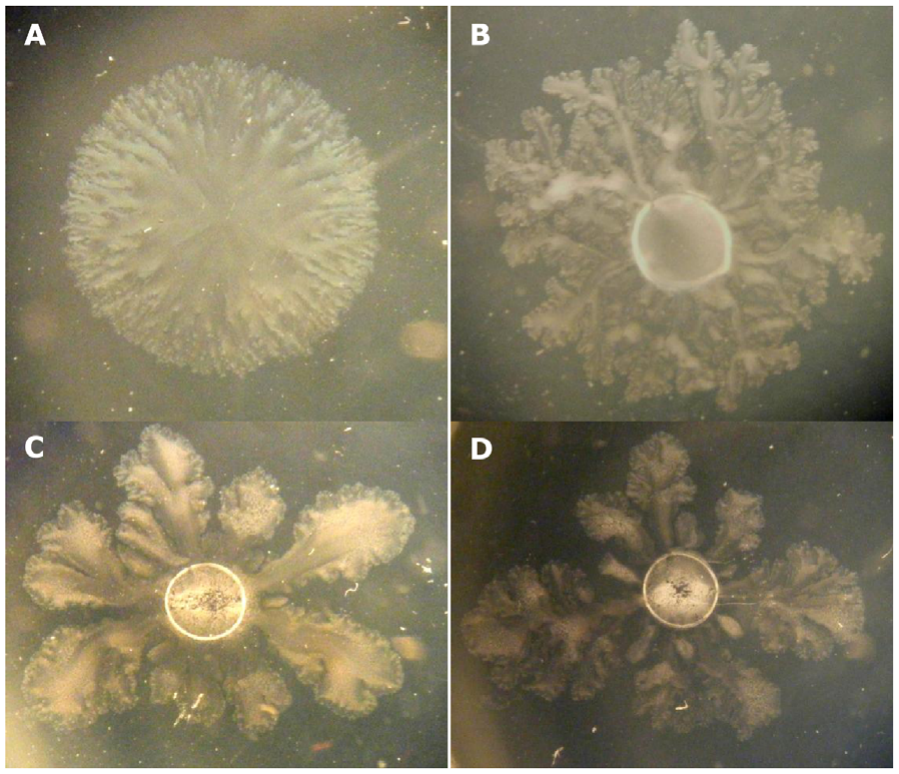
Swarms of *V. paradoxus* EPS imaged using a stereomicroscope. (A) wt+vec, (B) wt+*pil*Y1, (C) mut223+vec, (D) mut223+*pil*Y1.

Varpa_4680 and Varpa_5900 encode a putative L-fucosamine transferase (WbuB) and a putative PilY1 homolog, respectively. While all of the insertions into *wbu*B resulted in consistent phenotypes, different unique insertions in pilY1 resulted in varied phenotypes, including both increased and decrease biofilm and swarming responses (**Table 1**). Constructs containing each of these genes were introduced into wild type and mutant backgrounds, and compared to vector transformed strains. Overexpression of *wbu*B increased biofilm production in the wild-type background significantly, as did disruption of the gene in the chromosome (**Fig. 1A**). In the *wbu*B mutant background, complementation of both the biofilm and swarming defects was observed when the gene was supplied *in trans*. The swarm diameter at 48 h was only significantly different from wt in the insertion mutant transformed with vector alone, while in the biofilm assay the production of WbuB from the vector produced biofilms with the same density in wt and mutant backgrounds (**Fig. 1A, B**). The difference in phenotype between wt and mutant was also observed at the swarm edge microscopically (**Fig. 2A,C**) with the addition of the *wbu*B altering the mutant edge structure (**Fig. 2D**), increasing the apparent density and fluidity of the swarm. In the Varpa_5900 complementation strains, the altered biofilm phenotype was not restored by expression of a functional copy from a plasmid, but there was an increase in biofilm when *pil*Y1 was expressed *in trans* in either background (**Fig. 1C**). Expression of *pil*Y1 led to reduced swarming in both backgrounds at 48 h post-inoculation, resulting in no difference in swarm diameter between the wild-type and the complemented mutant. When we examined colony structure under the stereomicroscope, we saw that the expression of pilY1 *in trans* resulted in a wrinkled phenotype (**Fig. 3A, B**), as well as slower swarming. We also observed that the structure of the swarm was altered substantially in the mutant strain and that wild type structure was not restored in the complementation strain (**Fig. 3C, D**).

### Expression analysis of *wbu*B and *pil*Y1

**Figure 4:**
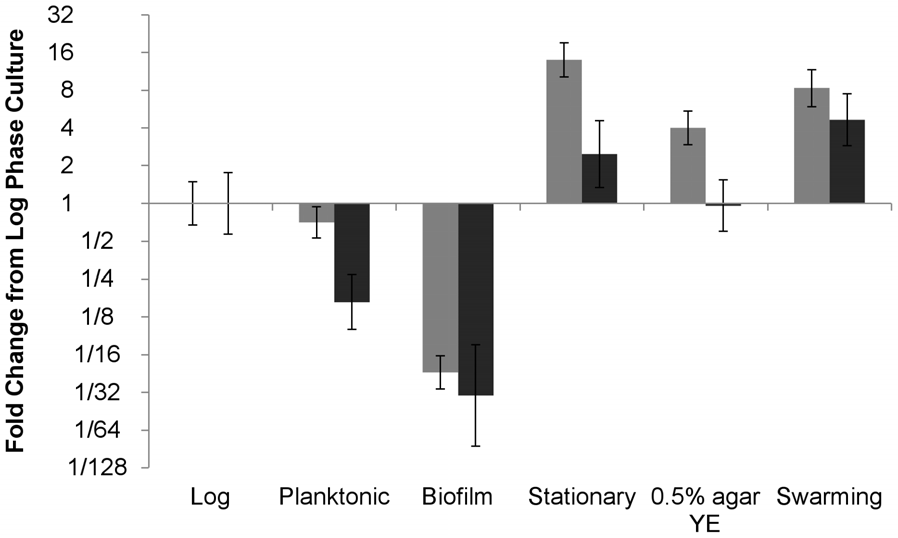
Expression of *wbu*B (grey bars) and *pil*Y1(black bars) assessed directly by qPCR. Planktonic cells, biofilm cells, and plate cultures from 0.5% agarose solidified YE plates or FW swarming plates were harvested at 48 h of growth and compared to aerated liquid culture in log phase (19 h) or stationary phase (26 h). RNA levels were determined in comparison to a luciferase spike added to each sample, and fold expression relative to log phase was assessed using the Pfaffl method.

When RNA was extracted from cultures grown in biofilms, on YE plates with 0.5% agar or on swarming plates, we observed substantial differences in both *wbu*B and *pil*Y1 RNA levels from log phase broth culture (**Fig. 4**). Expression levels in a given sample were normalized to an exogenous internal reference (firefly luciferase mRNA) added to the RNA samples as described previously [22]. We examined several potential internal reference genes (*gyr*A, *pro*C, *rps*L) as alternatives to the external spike, but found that we all of these genes had variable expression under the conditions examined (not shown). The expression of Varpa_4680 and Varpa_5900 was reduced compared to log phase growth in 48 h biofilms, and was increased after 48 h of swarming across a plate. Expression of both genes was also increased in stationary phase (26 h post inoculation, OD595 stable for ∼6 h, growth data not shown). The expression of these two genes diverged in the planktonic cells collected from the biofilm cultures, with only expression of *pil*Y1 diverging from log phase. When the bacteria were cultured on plates containing 2.5 g/L YE and 0.5% agarose, only expression of *wbu*B was increased relative to log phase.

#### ShkRS

**Figure 5:**
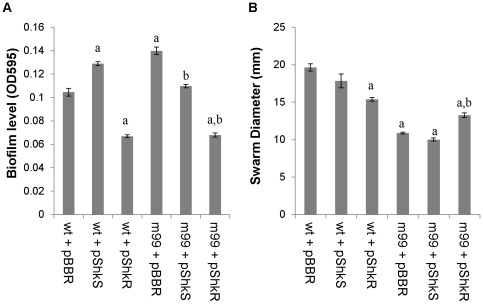
Analysis of surface behaviors to examine complementation of Varpa_4664 (*shk*S). Biofilm levels after 24 h incubation (A) and swarm diameters (B) at 48 h for wt and mut99 expressing either *shk*S or *shk*R *in trans* compared to vector controls. Error was computed as +/−SEM. P-values were calculated using the student's unpaired T-test. For both panels, a = p<0.01 compared to wt+vec, b = p<0.01 compared to mut99+vec.

Interruption of Varpa_4665 (*shk*S) increased the level of biofilm at 24 h and strongly delayed the onset of swarming motility (**Table 1**). Because this gene is thought to be part of a two component system with Varpa_4664 (*shk*R), each of these genes was used to individually complement a transposon insertion in *shk*S (mut99), with markedly different results. Biofilm formation was repressed by the presence of *shk*R in either mut99 or wt background, while *shk*S increased the level of biofilm in the wt background, and complemented the mutant (**Fig. 5A**). Expression of *shk*R significantly reduced swarming in the wt background, and was sufficient to significantly increase swarming in mut99, but not back to wt levels (p<0.01 for all comparisons) at 48 h (**Fig. 5B**).

## Discussion

### Exopolysaccharide synthesis

Our results from a transposon library screen using two separate criteria – colony morphology and biofilm phenotype – led to the identification of insertions in two loci predicted to encode exopolysaccharide (eps) synthesis and export functions (Varpa 1090-1098, Varpa4673-4690). The majority of the insertions were in the second putative eps synthesis region, much of which (Varpa4677-4685) is not present in *V. paradoxus* S110. The association of eps synthesis with biofilm formation has been shown in other species, and is not a surprising result. Polar effects on Varpa_4680 were observed when complementation of Varpa_4681 (encoding a putative epimerase), which limits our ability to determine the contributions of the disrupted genes. Future experiments using markerless deletions in the chromosome of the genes in these loci will allow for further examination of their roles in eps production and surface behaviors.

### WbuB

**Figure 6:**
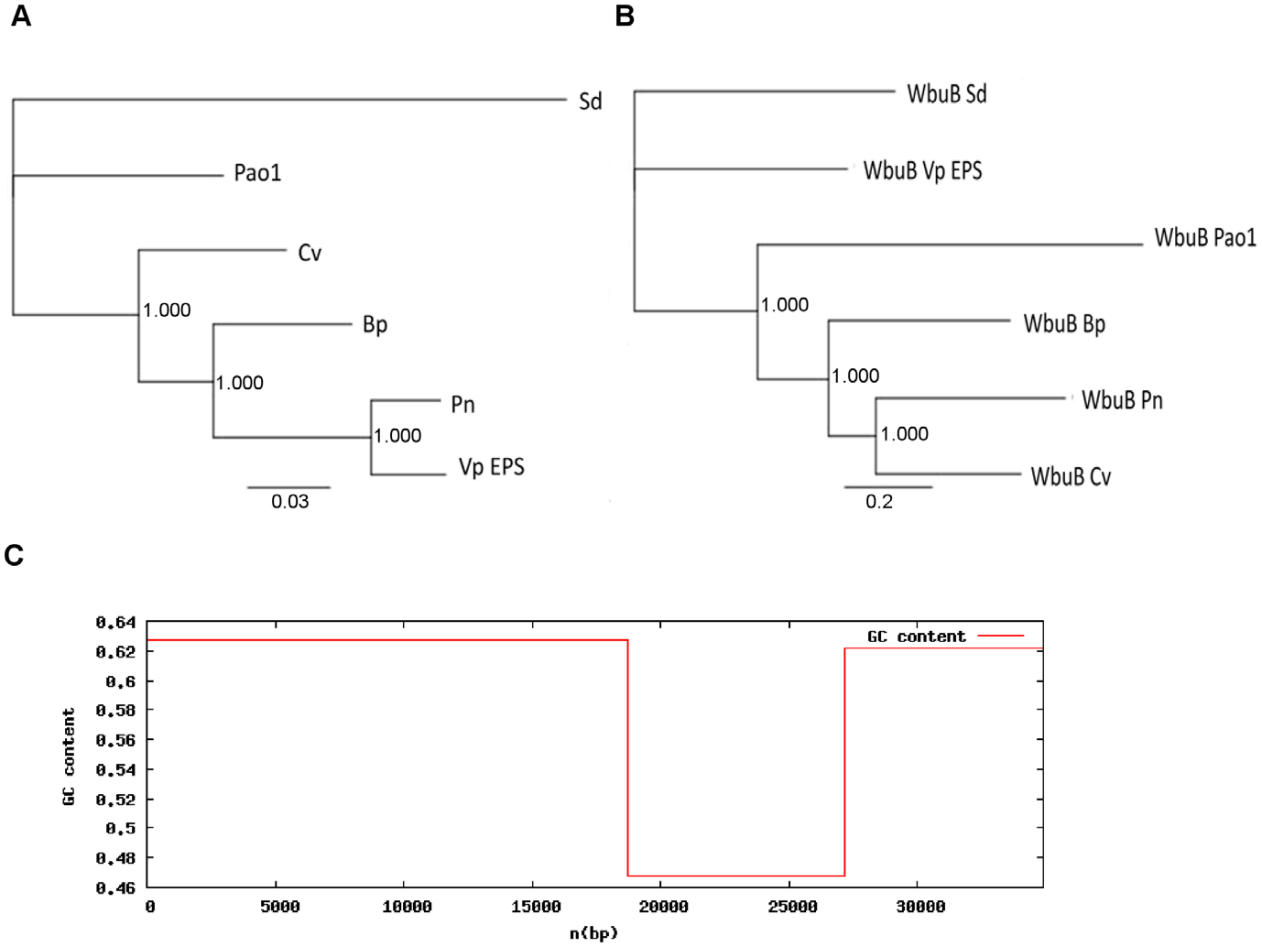
Phylogenetic and nucleotide composition suggests Varpa_4680 entered the *V. paradoxus* EPS genome by horizontal transfer. A) Phylogenetic tree based on WbuB amino acid sequences created using ClustalW multiple sequence alignment and subsequent Bayesian inference of phylogeny(Mr. Bayes). B) Phylogenetic tree using the 16s rDNA sequences from the same set of organisms as in (A), using Bayesian inference of phylogeny based on the HKY85 nucleotide substitution model. For both A) and B) Vp EPS = *Variovorax paradoxus* EPS, Pn = *Polaromonas naphthlenivorans* CJ2, Bp = *Bordatella pertussis* Tohama 1, Pao1 = *Pseudomonas aeruginosa* PAO1, Cv = *Chromobacterium violaceum* ATCC 12472, Sd = *Sulfurimonas denitrificans* DSM 1251 C) G/C analysis of 35 kb region of the *V. paradoxus* EPS genome including *wbu*B. Total region spans orfs Varpa_4661-4691. Low G/C region from 18.4 kb to 27.1 kb spans orfs Varpa_4679-4684.

The most frequently identified interrupted gene, *wbu*B (Varpa4680), encodes a putative N-acetyl-l-fucosamine transferase. Because of the two-layer screening technique employed in this work, this gene is by definition strongly influencing both colony morphology and biofilm formation. In *E. coli* this gene is involved in the synthesis of the O-antigen, but based on genomic location of Varpa_4680 it appears not to be part of the LPS synthesis locus. Comparison of the WbuB protein based tree of orthologs with the 16s rRNA tree derived from the same organisms (**Fig. 6A,B**), along with the GC content data for the eps locus and the lack of orthologs in the closest relative (**Fig. 6C**), suggests that the gene was acquired through horizontal transfer. Transposon mutant colonies on YE agar was considerably less mucoid, while overexpression of *wbu*B in this background resulted in restoration of the mucoid phenotype (not shown). Taken in combination with the complementation and gene expression data presented, it appears that WbuB is crucial to the production of a hygroscopic exopolysaccharide molecule involved in movement across surfaces, and inhibitory to the formation of biofilm. Work in *Thauera* and *P. aeruginosa* has identified N-acetyl-l-fucosamine is found in the eps of some bacteria [23] [24]. While in many organisms exopolysaccharides have been shown to be essential for biofilm formation, there are also instances where specific eps components such as cellulose may negatively impact this phenotype [25].

### PilY1

The second most frequently interrupted gene in our screen was Varpa_5900, which encodes a putative PilY1 protein that is hypothesized in other organisms to play both structural and regulatory roles in type IV pilus (tfp) formation [26] [27]. The tfp genes are spread throughout the genome of *V. paradoxus* EPS, as in other gram negative species [28]. No other tfp genes aside from Varpa_5900 were identified in this screen, suggesting that the identified phenotype is not directly related to type IV pilus formation. Expression *in trans* has an effect on both phenotypes tested, but cannot completely complement the defect. Based on operon prediction, it is unlikely that the phenotype is due to a polar effect. .The insertion mutant we used in our complementation studies is an interruption 1347 nt from the predicted translation start site (**Table 2**). It has been proposed that the N and C termini of PilY1 have different functions, so it is possible that the phenotype results from expression of an active truncated form. Creation of a deletion mutant will clarify these issues.

**Table 2:** Strains, Vectors, Primers.

*Name*	*Description*	*Source/other info*
***Bacterial Strains***		
*Variovorax paradoxus* EPS	Wild type strain collected at CSUSB	this study
*V. paradoxus* EPS mut86	Tn5 insertion in *wbu*B at nt24	this study
*V. paradoxus* EPS mut223	Tn5 insertion in *pil*Y1 at nt1347	this study
*V. paradoxus* EPS mut99	Tn5 insertion in *shk*S at nt413	this study
*E. coli* DH5α	F^−^ endA1 glnV44 thi-1 recA1 relA1 gyrA96 deoR nupG Φ80dlacZΔM15 Δ(*lacZYA-argF*)U169, hsdR17(r_K_^−^ m_K_^+^), λ–	[40]
*E. coli* S17-1λpir	*thi pro hsdR* ^−^ *hsd*M^+^ *recA* RP4 2-Tc::Mu-Km::Tn*7*(Tp^R^ Sm^R^) λ-pir	[41]
***Plasmids***		
pOT182	pSUP102::Tn5	[33]
pCR2.1	TA vector	Invitrogen
pBBR1MCS-2	Broad host range vector	[42]
pWbuB	pBBR1MCS2:Varpa_4680	This study
pPilY1	pBBR1MCS2:Varpa_5900	This study
pShkR	pBBR1MCS2:Varpa_4664	This study
pShkS	pBBR1MCS2:Varpa_4665	This study
***Primers***		
Erc2	ACAGATTTAGCCCAGTCGG	Sequencing rescue clones
Hgal	GTCAGCACCGTTTCTG	
Wbub n-term	CCATGGGTCTTTGGAGATGATTATCCA	*Nco*I at 5′ end
Wbub c-term	GAGCTCCCCCAATAGAACTGAGAAAT	*Sac*I at 5′ end
PilY1 n-term	AAATCCCATTTGAACCAGAG	
PilY1 c-term	GTTAACGACTGGACAGAGAAAAGG	
*shk*R forward	GAATTCCGCTGGAGGCATTGGCGGAA	*Eco*RI at 5′ end
*shk*R reverse	ACTAGTGACGAGGCGGTGGCACAGG	*Spe*I at 5′ end
*shk*S forward	GAATTCGCCGGTGCCGTCGAACTTGA	*Eco*RI at 5′ end
*shk*S reverse	ACTAGTCTGCACTACGCCCGCACCAA	*Spe*I at 5′ end
Lucfor	TACAACACCCCAACATCTTCGA	[22]
Lucrev	GGAAGTTCACCGGCGTCAT	[22]
Wbub for	GGCGTCTCTGTGGCCTGCG	
Wbub rev	GCGGCCAAATATACGCGCACC	
PilY1 for	GCAGCTCGAGCCTTGGCAACT	
PilY1 rev	CGCCCCTGTATAGCGCCGTG	

**Figure 7:**
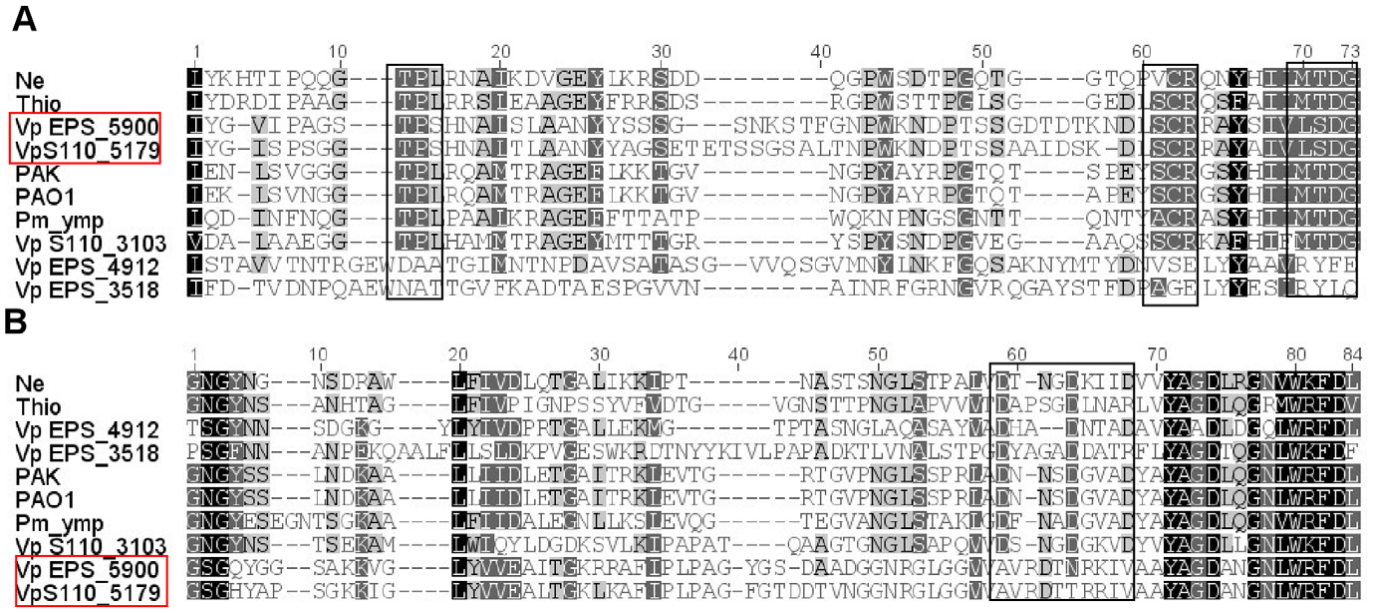
Conservation of putative active sites in three pilY1 homologs in *V. paradoxus* EPS. ClustalW alignment of PilY1 around (A) the N-terminal conserved site previously described in [29] and (B) the C-terminal Ca^2+^-binding site [27]. Varpa_5900 and its ortholog Vapar_5179 in *V. paradoxus* S110 are outlined in red, and the conserved functional sites in each region are outlined in black. Ne = *Nitrosomonas europaea* ATCC 19718 NE_1747,Thio = *Thioalkalivibrio sulfidophilus* HL-EbGr7 Tgr7_3225, Pm ymp = *Pseudomonas mendocina* ymp Pmen_0958, PAO1 = *Pseudomonas aeruginosa* PAO1 PA4554, PAK = *Pseudomonas aeruginosa* PAK PilY1.

There are two additional putative PilY1 alleles in *V. paradoxus* EPS. We aligned putative active sites from these three paralogs (Varpa_5900, Varpa_4912, Varpa_3518) with the sequences of orthologs from the *V. paradoxus* S110 and other species (**Fig. 7**). Based on previous work [26] we identified the putative N-terminal VWA region crucial to function as well as the Ca^2+^-binding C-terminal region [27]. The Varpa5900 *pil*Y1 gene has a only one variation at position×(TPS for TPL) in the conserved N-terminal region. Work in *P. aeruginosa* suggests that the threonine in this motif as well as the aspartate and glycine in the conserved MTDG, are critical to proper subcellular localization and control of swarming motility [29]. It remains to be seen if these residues play the same role in *V. paradoxus*. There is no evident conservation of the Ca^2+^-binding site in Varpa_5900. Neither of the other two putative *pil*Y1 alleles has significant conservation of the N-terminal active site, although a possible Ca^2+^-binding site is present in Varpa_4912. Interestingly, *V. paradoxus* S110 has a PilY1 allele encoded at Vapar_3103 that contains both the N-terminal and C-terminal conserved regions in better condition than any others in either strain. Thus a reasonable hypothesis to pursue in future work is that through gene duplication and diversification this organism has separated the functions of the N-terminal and C-terminal domains of the PilY1 protein in other microorganisms, and that this divergence postdates the divergence of these two *V. paradoxus* strains.

### Gene expression

Our results indicate that the regulation of *wbuB* and *pilY1* is coordinated, with each rising and falling relative to log phase liquid culture based on surface associated growth. This is counter-intuitive, since the observed phenotypes in the complementation experiments are opposed. One compelling hypothesis to explain this is that the role of pilY1 is regulatory rather than structural, and that it is controlling the expression of structural factors, possibly including WbuB. Experiments are currently underway to construct fusions of *pil*Y1 and *wbu*B promoters to fluorescent reporters, which will be used to determine when and where in the swarm or biofilm they are expressed. Clean knockouts are also clearly necessary to determine the roles of these genes accurately. These methods and others such as transcriptome sequencing are potentially useful for determining the genetic and molecular underpinnings of complex phenotypes.

### The ShkRS two component system

The data on the role of the ShkRS system is more difficult to interpret, and suggests a complex regulatory system. Presumably this two-component system (TCS) functions similarly to the well conserved and studied systems in other organisms. Because the genes are oriented in opposite directions in the genome, with promoters facing one another, a polar effect is unlikely in this instance, although other *cis* effects can't be excluded. The higher biofilm level in the *shk*S mutant and the sharply delayed swarming suggests that the system works by triggering the switch to a surface motile phenotype from a sessile one. The effects of the *shk*R construct are a possible result of cross talk between TCS when a response regulator is overexpressed, as observed in the EnvZ-OmpR system in *E. coli* [30]. This may not be biologically relevant, but this cross talk potential has recently been evaluated across many genomes computationally, and a substantial level of this activity (15–25% of RR phosphorylated by non-cognate sensors) is predicted [31]. The analysis of the ShkRS system using transcriptome sequencing in mutant and wild type backgrounds appears to be a straightforward followup experiment to examine this phenotype more closely.

### Other genes interrupted in the screen

A number of genes were identified in this screen in various facets of microbial life, such as secretion, autotrophy, and transcriptional regulation,.as well as genes with assigned function involved in the regulation of cyclic-di-GMP levels within the cell (**Table 1**). This small molecule second messenger is used in many organisms to integrate signals coming from different environmental signals to permit more effective microbial responses [32]. The roles of these signal transduction mechanisms will be of substantial importance in our future investigations.

### Conclusions

In this work we demonstrate that *V. paradoxus* EPS, a strain cultivated from the desert sage scrub rhizosphere, has a complex and highly regulated response to physical and nutrient conditions that lead to either surface adhesion or motility.. We have observed that two genes in particular, Varpa_4680 (*wbu*B) and Varpa_5900 (*pil*Y1) play fundamental roles in both phenotypes, and are coordinately regulated in both biofilm and swarming responses. Finally, we identify a two component system, *shk*RS, that has a strong impact on both of these surface associated phenotypes. Future work to understand this regulatory network and the roles of these phenotypes in the environment will shed light on the mechanisms by which *V. paradoxus* succeeds in the soil environment.

## Materials and Methods

### Bacteria used

*V. paradoxus* strain EPS was cultivated from the coastal sage scrub rhizosphere soil in the Land Lab at CSU San Bernardino. Several *Escherichia coli* strains were used for molecular manipulations as well (**Table 2**).

### Transposon library construction

Transposon insertion was accomplished through bi-parental mating experiments using *E. coli* S17-1 λpir (pOT182:Tn5) as the donor strain, and *V. paradoxus* EPS as the recipient strain [33]. The donor was grown in LB+10 mg/L Gm, the recipient was grown in YT broth. The cultures were mixed at 1% v/v each strain in 10 mM MgSO_4_, and the broth was filtered onto a 0.2 micron cellulose acetate membrane (sterile, Whatman) using a disposable filter top bottle under vacuum. The membrane was transferred to non-selective media (YT) supplemented with MgSO_4_. The culture grew for 24 h at 30°C, and was washed in PBS containing 10 mM MgSO_4_. The wash fluid was plated onto M9 minimal salts agar using agarose as the solidifying agent, 0.2% glucose as the carbon source, and 25 mg/L Tc to select for transposon insertion. S17-1λpir requires supplementation with proline and histidine, and therefore does not grow on this medium [34]. Mutants from these plates were scraped and resuspended in PBS+15% glycerol, and frozen in microfuge tubes in 1 ml aliquots at −80°C until screening. The random insertion of the transposon, and the lack of plasmid DNA was determined by rescue cloning and PCR experiments (not shown).

### Screening

Mutant stocks were diluted serially and grown on 1.5% agar plates containing either 0.5 g/L or 2.5 g/L YE. Unusual colony morphologies compared to wild-type *V. paradoxus* EPS plated in the same fashion were selected for further study.

### Static biofilm assay

Relative biofilm production levels were evaluated using the 96-well crystal violet assay, adapted from the protocol previously developed for *Pseudomonas aeruginosa* [21]. Cultures were grown overnight in the appropriate nutrient broth corresponding to screening conditions, diluted 1∶20 with fresh broth, then placed into round bottom 96 well plates (BD Biosciences). The wells at the outer edge of the plate were filled with 200 µL sterile distilled water to avoid dehydration. Each sample was plated in quadruplicate and each plate contained a wild type control. Endpoint growth analysis of these cultures was performed by optical density measurement (595 nm) to eliminate mutants with severe growth defects. Two identical plates were prepared for each sample set, and grown for 24 h at 30°C. In one plate, the media was exchanged after 24 h and the plate was allowed to grow for a total of 48 h. After staining and resolubilization in 95% EtOH, 125 µL was transferred to a flat-bottom microtiter dish, and the absorbance at 595 nm was determined using a Thermomax spectrophotometer (Molecular Devices). Divergence from wild type tested in the same experiment was assessed using the Student's unpaired T-test. Mutants with significant variation from wild type were verified in a second biofilm assay, and only mutants that repeatedly varied in the same manner were pursued further. Biofilm assays on strains containing complementation constructs were performed similarly, with 50 mg/L Km and 25 mg/L Tc added to the overnight culture medium where appropriate. The biofilm assay medium was not supplemented with antibiotic, and biofilms were measured after 24 h of incubation.

### Rescue cloning and gene identification

The rescue cloning of the interrupted genes in the mutants of interest was performed using the method previously described [33]. Genomic DNA from the Tn5 insertion mutants was extracted (Wizard SV, Promega) and digested using either *Eco*RI or *Hin*DIII restriction enzymes (Promega). Each of these enzymes recognizes and cleaves at a single site within the Tn5 transposon. T4 DNA ligase (Fisher Scientific) was added to the digestions, which were recovered in chemically competent *E. coli* by plating on LB agar containing 25 mg/L Tc. The recovered plasmids were verified by agarose gel electrophoresis. Sequencing was performed at the Microchemical Facility at San Diego State University (http://www.sci.sdsu.edu/dnacore/sdsu_dnacore.html). The sequencing primers to amplify the region captured during rescue cloning are created using sections of the transposon are erc2 for the *EcoRI* digested rescue clone and Hgal for the *HinDIII* rescue clones (See **Table 2**). Sequences obtained from this effort were manually curated using Geneious Pro v3.7 (Biomatters, Ltd, New Zealand) and compared to the *V. paradoxus* EPS genome (http://img.jgi.doe.gov/) for insertion site identification.

### Electroporation

The procedure for introducing plasmid DNA by electroporation into *V. paradoxus* EPS was adapted from a protocol for *Pseudomonas* [35]. An overnight culture was diluted 1∶100 into 500 ml YE broth and growing to OD595 between 0.3 and 0.5 measured using a Spec20D spectrophotometer. To improve the efficiency of uptake, the prepared culture could be stored at 4°C for up to 48 h. High density cell suspensions in 300 mM sucrose were electroporated using a BioRad micropulser (Biorad Inc, Hercules CA) using 2 µL of DNA sample and 100 µL of cells in a 0.2 mm gap cuvette. After electroporation, 900 µL YE media was added and the cells were allowed to recover at 30°C for approximately 2 hours. The cells were plated on YE agar containing the appropriate antibiotics (25 mg Tc and/or 50 mg Km per L)

### Swarming Motility

Mutants that were previously screened for biofilm formation and were determined to have significantly altered biofilm phenotypes were screened altered swarming motility on freshwater medium (FW) plates [8] containing 0.2% glucose and 0.1% casamino acids. The bacteria were grown overnight in 2.5 g/L YE and washed in FW medium (no C source). For plating, they were resuspended at OD595 = 1 and 5 µL of the culture was plated in triplicate. The diameters of the colonies were measured and photographs were taken, both of the overall colony structure as well as the colony's swarming edge using 100× total magnification under phase contrast (Zeiss Axioplan 2). Images were captured with a CoolSNAP FX camera controlled using Imagepro Express v. 2.0. Plates were air dried for 4 days before using, and all comparative experiments were done using identical plates. For analysis of complementation constructs, Km was added (50 mg/L broth, 25 mg/L agar) and endpoint swarming diameters were measured at 48 h.

### Complementation

The genes to be tested were amplified using primers designed either from rescue cloned sequences or from the *V. paradoxus* EPS draft genome sequence. The products were cloned into pCR2.1 using the TopoTA cloning system (Invitrogen), with appropriate restriction sites added to the ends for subsequent cloning into pBBR1MCS2 (KmR). The subcloned products were verified by gel electrophoresis and subsequently transferred to the appropriate *V. paradoxus* strain by electroporation, as described above. The list of primers is in **Table 2**. Flanking DNA was included to allow for expression of all cloned genes from their native promoters. Each gene of interest was transformed into a Tn5 mutant strain (m86:*wbu*B, m223:*pil*Y1, m99:*shk*S) as well as into the wild type. Each mutant strain was also transformed with the vector alone, and all of these strains were compared to the wild type transformed with pBBR1MCS2 for complementation analysis.

### *In silico* analysis

Genes with high protein sequence similarity to *wbu*B were identified using BlastX [36], and multiple sequence analysis was performed. The phylogenetic tree derived from the structural gene was compared to 16S rDNA based tree. The WbuB protein tree was built using clustalw alignments validated using ProtTest, and Mr. Bayes was used to generate the tree. The corresponding 16S tree was generated using the HKY85 nucleotide substitution. Both trees were generated using 1,000,000 iterations with a 10,000 test burn in. Mr Bayes and clustalw were run in the GeneiousPro 3.1.7 genomic analysis program. Analysis of G+C content of the eps region and surrounding chromosome was performed using GC-Profile [37] (http://tubic.tju.edu.cn/GC-Profile/) with default parameters. The PilY1 protein sequences were derived from the finished genomes of the two strains of *V. paradoxus* S110 and EPS, along with selected PilY1 proteins based on BlastX searches with Varpa5900 (*Nitrosomonas europaea*, *Pseudomonas mendocina* ymp, *Thioalkalivibrio sulfidophilus* HL-EbGr7) and the two *P. aeruginosa* strains, PAK and PAO1, for which either structural or genetic analysis had identified important functional regions [29] [27].

### Gene expression analysis by qPCR

For the plate based assays, bacteria inoculated from a fresh overnight culture were spotted onto either swarming plates or plates containing 0.5% agarose and 5 g/L yeast extract; the bacteria under the latter condition do produce an exudate containing wetting agent (not shown) but do not swarm. All plate cultures were grown for 48 h at 30°C before harvesting. Cells were harvested in 1 ml sterile water using a sterile glass spreader. A set of eight plates was harvested for each sample in this manner. Biofilms were grown for 24 h as before, but 12 well culture plates were used rather than microtiter dishes. Planktonic cells from the biofilm culture were collected in 1 ml aliquots from the culture dishes. Biofilm samples were collected by aseptically scraping the cells from the walls and suspending them in sterile water. The cells collected from four different wells were used for each sample. The optical density of a liquid culture in 2.5 g/L YE was measured over a time-course during growth with shaking (200 rpm) at 30°C, and samples were harvested from the culture during log phase (18 h p.i.) and stationary phase (26 h p.i.). RNA was purified from all cell collections using the SurePrep True Total RNA Purification kit (Fisher) with the added DNase treatment step. Promega GoScript Reverse Transcriptase was used to make cDNA starting with 300 ng of sample RNA. To control for variation in RT efficiency, 100 pg of firefly luciferase RNA was added to each RNA sample before reverse transcription [22]. The cDNA samples were diluted 1∶10, aliquoted and stored at −20°C until analysis. The qPCR reactions were carried out in an Mx3005p (Stratagene) using the ABsolute Blue QPCR SYBR Green mix Plus ROX(Fisher). The primers were developed for the *V. paradoxus* EPS genes of interest using Primer3 [38], and these primers or luciferase specific primers (**Table 2**) were added to a final concentration of 70 nm. Each qPCR reaction was performed in triplicate using 3 µLof the cDNA for each reaction, and each test was repeated twice for each gene. The entire experiment was repeated three times using fresh cultures. The relative amounts of *wbu*B and *pil*Y1 message were calculated using the luciferase standard, to account for RT efficiency and qPCR reactions, and a comparator log phase sample using the Pfaffl method for comparative transcript analysis [39]. Efficiencies of the PCR reactions were calculated based on standard curves for each primer pair. The consistency of log phase culture gene expression was validated by measuring the expression of *wbu*B and *pil*Y1 in this manner from triplicate log phase cultures.
